# Editorial: Machine learning in radiation therapy for lung cancer

**DOI:** 10.3389/fonc.2024.1444543

**Published:** 2024-07-02

**Authors:** James C. L. Chow, Tonghe Wang

**Affiliations:** ^1^ Radiation Medicine Program, Princess Margaret Cancer Centre, University Health Network, Toronto, ON, Canada; ^2^ Department of Radiation Oncology, University of Toronto, Toronto, ON, Canada; ^3^ Department of Medical Physics, Memorial Sloan Kettering Cancer Center, New York, NY, United States

**Keywords:** artificial intelligence (AI), lung cancer, radiotherapy, deep learning - artificial intelligence, machine learning, adaptive radiotherapy (ART), NSCLC, big data & analytics

The integration of machine learning (ML) into radiotherapy (RT) for lung cancer marks a significant leap forward in the field of oncology. As the burden of lung cancer continues to rise globally, there is an urgent need for innovative approaches that can enhance the precision, effectiveness, and personalization of treatments. This research topic of Frontiers in Oncology, titled “Machine Learning in Radiation Therapy for Lung Cancer,” brings together cutting-edge research that demonstrates the transformative potential of ML technologies. By leveraging advanced algorithms and large datasets, these studies aim to optimize treatment planning, improve predictive accuracy, and ultimately, enhance patient outcomes. This collection of papers not only highlights the current advancements but also sets the stage for future innovations in the integration of ML into lung cancer RT. Highlights include the use of deep learning to enhance adaptive RT and a bibliometric analysis on ML in non-small cell lung cancer (NSCLC) RT. Moreover, the research topic contains work demonstrating the efficiency of automated treatment planning through reinforcement learning, and a study assessing the interfraction stability of the delivered dose distribution by exhale-gated volumetric modulated arc therapy (VMAT) or intensity-modulated arc therapy (IMAT) for lung cancer. Furthermore, there is research exploring the predictive power of ML in assessing the risk of radiation pneumonitis.

Hooshangnejad et al. present a study on the implementation of a novel, deeply accelerated adaptive RT (DAART) approach for lung cancer RT. Given that lung cancer remains the leading cause of cancer-related deaths, and RT is a crucial treatment for medically inoperable early-stage NSCLC, the study of Hooshangnejad et al. addresses a critical challenge in reducing the time from diagnosis to treatment initiation. The current median time of four weeks can lead to restaging and loss of local control, but the DAART approach, featuring the innovative deepPERFECT system, aims to significantly shorten this delay. Zhang et al. conducted a comprehensive bibliometric analysis to investigate the progress, research trends, and hotspots in the application of ML to RT for NSCLC. As ML becomes increasingly integrated into NSCLC RT, understanding these trends is crucial for guiding future research and development. The study of Zhang et al. provides valuable insights into the current state of ML applications in NSCLC RT and highlights potential hot areas for future research, thereby aiding researchers in identifying emerging trends and opportunities in this field. Moreover, Wang et al. present a novel integrated solution for automatic intensity-modulated radiation therapy (IMRT) planning in NSCLC cases. This study aims to enhance the efficiency and consistency of treatment planning using advanced ML techniques. Wang et al. demonstrates the feasibility and potential of this integrated solution to streamline the planning workflow and reduce variation in plan quality across different regions and treatment centers, paving the way for further improvements and broader clinical implementation. Guberina et al. present a study aimed at assessing the interfraction stability of the delivered dose distribution in exhale-gated VMAT or IMAT for lung cancer. This study also seeks to identify dominant prognostic dosimetric and geometric factors influencing treatment effectiveness. The study of Guberina et al. demonstrates that accumulated dose distributions over treatment series are robust against interfraction CTV deformations when using exhale gating and online image guidance. D_min_ was identified as the most critical parameter for predicting gEUD in a single fraction. Other geometric parameters provided limited additional predictive value. These findings underscore the importance of dosimetric information, particularly the location and value of D_min_ within the CTV_i_, for optimizing image-guided radiation treatment. In addition, Ye et al. have developed an optimal ML model to predict the occurrence of radiation pneumonitis (RP) in lung cancer patients treated with VMAT. This study emphasizes the utility of lung equivalent uniform dose (lung EUD) as a predictive metric for RP, aiming to enhance predictive accuracy and treatment planning. The study of Ye et al. utilized four prominent machine learning algorithms, demonstrating that lung EUD-based factors substantially enhanced predictive performance for RP 2+. The results advocate for the decision tree model with lung EUD-based predictors as the optimal tool for predicting RP in VMAT-treated lung cancer patients, potentially replacing conventional dosimetric parameters and simplifying complex neural network structures in prediction models.

The collection of papers featured in this research topic presents a transformative shift in the lung cancer treatment. Each study addresses critical challenges within the field, ranging from accelerating adaptive RT to predicting radiation pneumonitis occurrence. These advancements signify a significant step forward in optimizing treatment planning, enhancing precision, and improving patient outcomes. By harnessing the power of ML algorithms, researchers have developed innovative solutions that streamline treatment workflows, reduce planning uncertainties, and enable personalized care for patients with lung cancer. Furthermore, the integration of advanced dosimetric parameters and predictive models offers clinicians valuable insights into treatment response and toxicity prediction, ultimately guiding more informed decision-making processes.

In contemplating the future direction of RT for lung cancer, the integration of ML is poised to play a pivotal role. As demonstrated by the studies featured in this research topic, ML algorithms hold immense potential in optimizing treatment planning, predicting treatment outcomes, and personalizing patient care. Looking ahead, further research in this domain is anticipated to focus on refining existing models, expanding datasets, and integrating multi-modal data sources to enhance predictive accuracy. Moreover, efforts towards the development of automated treatment planning systems and real-time adaptive strategies are expected to accelerate, aiming to streamline clinical workflows and mitigate uncertainties during treatment delivery. Furthermore, the integration of artificial intelligence and deep learning techniques offers promising avenues for novel insights into tumor biology, treatment response, and patient prognosis. Collaborative efforts between clinicians, physicists, and data scientists will be paramount in translating these technological advancements into tangible clinical benefits. Additionally, it is imperative to maintain a steadfast commitment to patient-centered care and ethical considerations, ensuring that these transformative technologies are harnessed responsibly to improve patient outcomes and quality of life.

## Author contributions

JC: Writing – original draft, Writing – review & editing. TW: Writing – review & editing.

